# Trends in urban/rural inequalities in physical growth among Chinese children over three decades of urbanization in Guangzhou: 1985–2015

**DOI:** 10.1186/s12889-020-09239-7

**Published:** 2020-07-31

**Authors:** Yan Hu, Weiqun Lin, Xuying Tan, Yu Liu, Yuqi Wen, Yanfei Xing, Ying Ma, Huiyan Liu, Yanyan Song, Jingjing Liang, Kin Bong Hubert Lam, Suifang Lin

**Affiliations:** 1 Department of Children Healthcare, Guangzhou Women and Children’s Medical Center, Guangzhou Medical University, Guangzhou, 510623 People’s Republic of China; 2 Department of Clinical Nutrition, The First Affiliated Hospital of Guangdong Pharmaceutical University, Guangzhou, 510080 People’s Republic of China; 3 Department of Medical Statistics and Epidemiology, School of Public Health, Sun Yat-sen University, Guangzhou, China; 4 Nuffield Department of Population Health, University of Oxford, Oxford, UK

**Keywords:** Health inequalities, Physical growth trend, Economic reform, Guangzhou city

## Abstract

**Background:**

Great growth inequalities between urban and rural areas have been reported in China over the past years. By examining urban/rural inequalities in physical growth among children < 7 years old over the past three decades from 1985 to 2015 in Guangzhou, we analyzed altering trends of anthropometric data in children and their association with economic development during the period of rapid urbanization in Guangzhou.

**Methods:**

The height, body weight and nutrition status of children under 7 years old were obtained from two successive cross-sectional surveys and one health surveillance system. Student’s *t*-test, Spearman’s rank-order correlation and polynomial regression were used to assess the difference in physical growth between children in urban and rural areas and the association between socioeconomic index and secular growth changes.

**Results:**

A height and weight difference was found between urban and rural children aged 0–6 years during the first two decades of our research (1985–2005), which gradually narrowed in both sex groups over time. By the end of 2015, elder boys (age group ≥5 year) and girls (age group ≥4 year) in rural areas were taller than their counterparts in urban areas (*p* < 0.05).The same trend could be witnessed in the weight of children aged 6 years, with a − 1.30 kg difference (*P* = 0.03) for boys, and a − 0.05 difference (*P* = 0.82) for girls. When GDP increased, the gap in boys’ weight-for-age z-score (WAZ from 0.25 to 0.01) and height-for-age z-score (HAZ from 0.55 to 0.03) between urban and rural areas diminished, and disappeared when the GDP per capita (USD) approached 25,000. In either urban or rural areas, the urbanization rate and GDP were positively associated with the prevalence of obesity (all *R* > 0.90 with *P* < 0.05) and negatively correlated with the prevalence of stunted growth (all *R* < -0.87 with *P* < 0.05).

**Conclusion:**

Growth inequalities gradually decreased with economic development and urbanization, while new challenges such as obesity emerged. To eliminate health problems due to catch-up growth among rural children, comprehensive intervention programs for early child growth should be promoted in rural areas.

## Background

Height and weight are important indicators in measuring children’s physical growth and development [1]. They not only reflect nutrition and health condition in children, which is related to their socioeconomic status, but also influence their well-being and quality of later life [2, 3]. Investigating the secular trend in physical growth for 0- to 7- year-old children reveals the patterns of child growth and thus, provides valuable biological evidence for policymakers to develop public health strategies [2].

Since the 1950s, several studies have compared the secular growth trends between girls and boys in China’s urban and rural areas [4, 5]. Over the past three decades, rapid economic development and enhanced living standards have enabled positive secular trends in children’s physical growth. Anthropometric measurements have become crucial for assessing socioeconomic status [4]. However, the significant regional, east-west and socioeconomic disparities in China and their effect on children’s physical growth cannot be ignored [6–8]. Under the economic reform in China, Guangzhou, as a pioneer of this economic opening, has been through rapid urbanization and economic development [9, 10]. At the same time, income inequalities have emerged, along with an increase in health inequalities [11]. Limited studies have tried to link inequalities of children’s growth to dramatic socioeconomic changes, which acts as important evidence for the government to develop policies that improve early childhood development and health care [2, 12]. With the fast economic development in China, how inequalities in child growth development have changed over the past years in urban and rural areas of Guangzhou remains unclear, and further study is needed to estimate the magnitude of differences in urban and rural areas.

The present study investigated the influence of economic development in health inequalities. The goal of this study was to describe inequalities in child growth and its trend from 1985 to 2015 in Guangzhou. We focused on assessing 1) the difference in urban/rural child growth rate and its change over the past 30 years, 2) whether growth inequalities have disappeared under urbanization in Guangzhou, and 3) the association between economic development and growth inequality.

## Methods

### Data sources

Data used to estimate the growth and nutritional status of children aged < 7 years were extracted from cross-sectional surveys, including: National Survey on the Physical Growth and Development of Children in the Nine Cities in China (NSPGDC) [13], National Epidemiology Survey on Simple Obesity in Childhood (NESSOC) [14], China National Maternal and Child Health Surveillance System (MCHSCN). A summary of these surveys were presented in Supp. Table 1. The study was approved by the Ethics Committees of Capital Institute of Pediatrics (SHERLL 2015009).

### Growth data

Growth data were included from the second to the fifth NSPGDC [15, 16]. The surveys were conducted in 1985, 1995, 2005 and 2015 respectively. As a national research site, we collected children’s physical growth data in Guangzhou.

We used a multi-staged, stratified cluster-sampling method following the NSPGDC guideline. First, we stratified subjects by urban (Yuexiu, Liwan and Haizhu Districts) and rural (Conghua, Huadu, Panyu and Baiyun Districts) areas based on the level of socioeconomic development. The partitioning of urban and rural areas was cited from a previous research which used a location quotient to reflect regional characteristics, including gross regional product per unit area, population density, non-agricultural population ratio and etc., of a specific area in Guangzhou [17].

Next, we sampled hospitals (for infants under 1 month of age), neighborhood communities (for children aged 1 month to 3 years) and kindergartens (for children aged 3 to 6 years) in corresponding areas as the cluster units. Participants with the following conditions were excluded: a) infants born before 37 weeks of gestation or weighed less than 2.5 kg at birth, b) twins or other multiple births, c) children with congenital, endocrine, neurological or chronic systemic diseases, d) children who have had a fever for more than 7 days within the past 2 weeks, e) continuous diarrhea more than 5 times/day for no less than 5 days.

Children were grouped by sex (male/female), age (at birth, 1 month, 2 months, 3 months, 4 months, 5 months, 6 months, 8 months, 10 months, 12 months, 15 months, 18 months, 21 months, 2 years, 2.5 years, 3 years, 3.5 years, 4 years, 4.5 years, 5 years, 5.5 years and 6–6.9 years) and region (urban/suburban). Consequently, we included a total of 67,545 children, and each sex/age subgroup of urban/suburban areas included 150–200 children (Supplementary Table 1).

Two well-trained investigators collected anthropometric data from each child. Height was measured without shoes and rounded to the nearest 0.1 cm (the length was measured for children aged < 3 years). Their weight was measured with light clothing and rounded to the nearest 0.05 kg following standard techniques described in previous research [15]. Each measurement was repeated three times with means rounded to the nearest 0.1 cm.

### Obesity and stunted growth data

We obtained data on the prevalence of obesity from 1996 to 2015 in NESSOC. The NESSOC was conducted approximately every 10 years. All children aged < 7 years who lived in administrative urban areas (Yuexiu and Haizhu Districts) or rural areas (Panyu and Huadu Districts) in 2006 were included in this study. The total sample size ranged from 6000 to 8000 (Supplementary Table 1). Data on the prevalence of stunted growth among children aged < 7 years were obtained from the MCHSCN. We gathered data from nearly 800,000 children per year.

Obesity was defined using weight for height index for 0–2-year-old children or body mass index for children older than 2 years. Children were categorized as obese if their corresponding indicators were > 3 standard deviations (SD) from the mean level according to the standardized growth charts from the Working Group on Obesity in China [18]. Stunted growth was defined by length or height for age (LA or HA < -2 z-score) based on the WHO Child Growth Standard (2006).

### Economic data

Indicators of economic development (gross domestic product [GDP] per capita and urbanization rate) in Guangzhou were obtained from the Statistical Yearbook of the Guangzhou Statistics Bureau [19].

### Data analysis

We calculated means and SDs for continuous variables and percentages for categorical variables. Absolute growth differences were calculated according to the following formula:

$$ \mathrm{Growth}\ \mathrm{difference}={G}_{urban}-{G}_{rural} $$

where *G*_*urban*_ and *G*_*rural*_ represent the mean height or weight for urban and rural children, respectively. To identify and compare temporal changes of growth patterns in urban and rural children, height/weight z-scores adjusted for age and sex (HAZ/WAZ) were calculated using the Chinese national growth standards of 2005 [20] with the formula:

$$ z- score=\frac{x-\mu }{\sigma } $$

where *x* is the mean measurement for age, μand σ is the age- and sex-specific reference mean and standard deviation for *x*.

Degree of urbanization was calculated by the proportion of the non-agricultural population. Student’s *t*-test were used to compared height and weight between rural and urban groups. Prevalence of obesity and stunted growth were assessed using a chi-square test. Spearman’s rank-order correlation coefficients were used to analyze the correlation between GDP per capital and urbanization rate and the prevalence of obesity and stunted growth. *P* value < 0.05 was defined as statistically significant. Polynomial regression was used to analyze the trend correlation between GDP per capital and HAZ/WAZ. All data analyses were performed using R software version 3.5.1 (https://www.r-project.org/).

## Results

### Urban/rural height and weight differences in Guangzhou

**Fig. 1:**
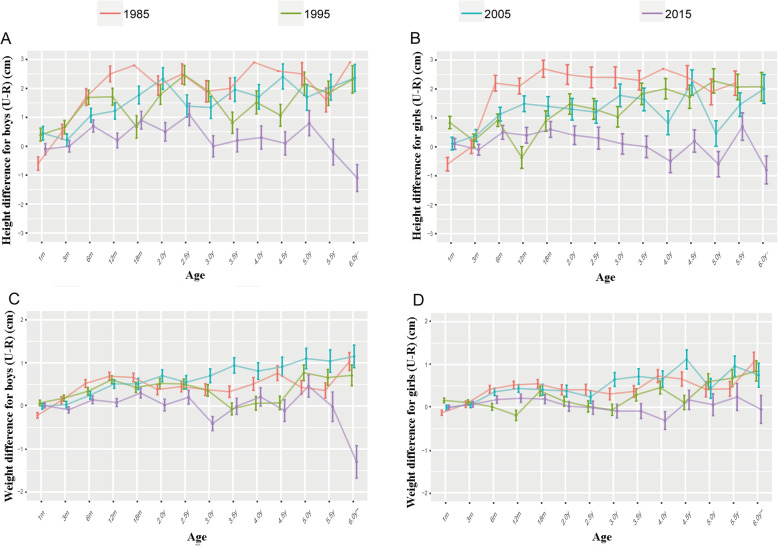
Regional height*/weight differences of children between Urban and Rural areas in Guangzhou. **a** Height difference for Boys. **b** Height difference for Girls. **c** Weight difference for Boys. **d** Weight difference for Girls. Values are shown as the mean ± SD. For more detailed information (difference and *p* value), refer to Supplementary Table 4 and 5. *Height was measured as the length of children < 3 years of age

Urban/rural differences in growth were calculated by the formula listed in the methods section, and further analyzed by the Student’s *t*-test. As shown in Fig. 1 and Supplementary Fig. 1, urban children were significantly taller than their rural counterparts in most age groups during the first 2 decades (1985–2005). However, height differences between urban and rural children have gradually narrowed in both sex groups over time. By the end of 2015, elder boys and girls (boys aged 5, 5.5 and 6 years and girls aged 4, 4.5, 5, 5.5 and 6 years) in the rural area were taller than their counterparts in the urban areas (*P* < 0.05, supplementary Table 2). Interestingly, a fluctuating trend was observed in urban/rural weight difference as shown in Fig. 1c. The gap between areas became small during the first decade (1985–1995), but then it re-emerged between 1995 and 2005. Again, the gap narrowed in 2015. In 1985, the weight difference of 6-year-olds between urban and rural areas was 1.03 kg (95%CI: 0.62, 1.44, *P* = 0.04) for boys and 1.06 kg (95%CI: 0.61, 1.51, *P* = 0.04) for girls. However, by the year 2015, the weight of boys in rural areas exceeded that of urban areas in the same age group, with a − 1.30 kg difference (95%CI: − 2.04, − 0.56, *P* = 0.03, supplementary Table 3), while the weight of the girls in rural and urban areas were basically the same (difference: -0.05, 95% CI: − 0.70, 0.60, *P* = 0.82). These results showed a significant change in the physical growth inequalities between urban and rural children in Guangzhou.

### Inequalities in growth z-score and their association with economic development in Guangzhou

**Fig. 2:**
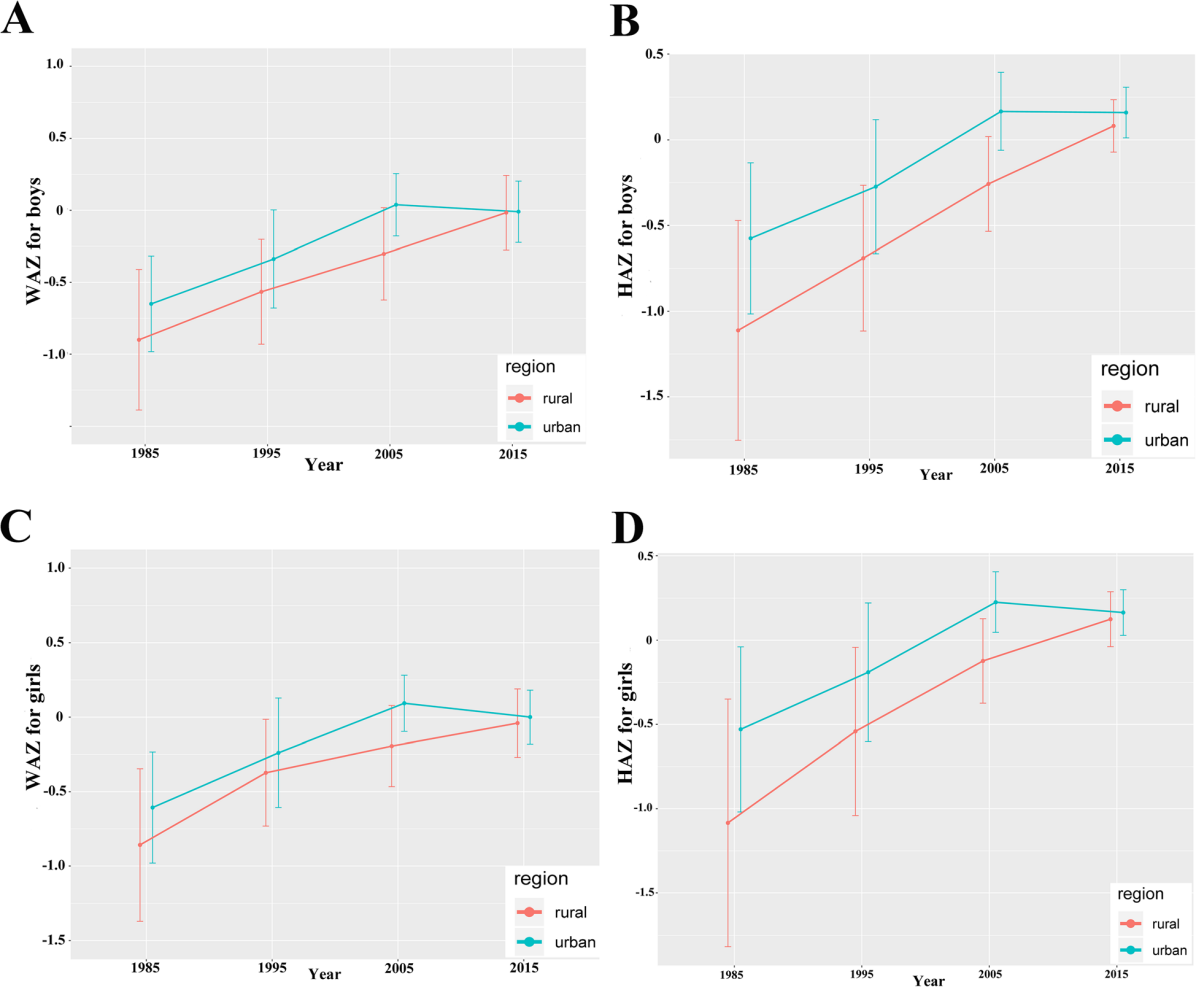
HAZ and WAZ of children between Urban and Rural areas in Guangzhou, 1985–2015. **a** WAZ for boys. **b** HAZ for boys. **c** WAZ for girls. **d** HAZ for girls. Values are shown as the mean ± SD

To assess the inequalities of physical growth in urban and rural children across a diverse age group over the past 30 years, HAZ and WAZ in each sex group were computed and further analyzed by the student’s *t*-test. HAZ and WAZ in different areas and their difference were showed in Fig. 2. Both HAZ and WAZ of urban children had been on a persistent upward trend between 1985 and 2005 but had been on a slight downward trend since 2005. For rural children, a continuous upward trend was observed in both height and weight since from 1985 to 2015. However, the gap in WAZ and HAZ between boys from urban and rural areas diminished (from 0.25 to 0.01 and from 0.55 to 0.03, respectively). The same change was also observed in girls, with the difference in WAZ from 0.25 to 0.04 and HAZ from 0.55 to 0.04.

**Fig. 3:**
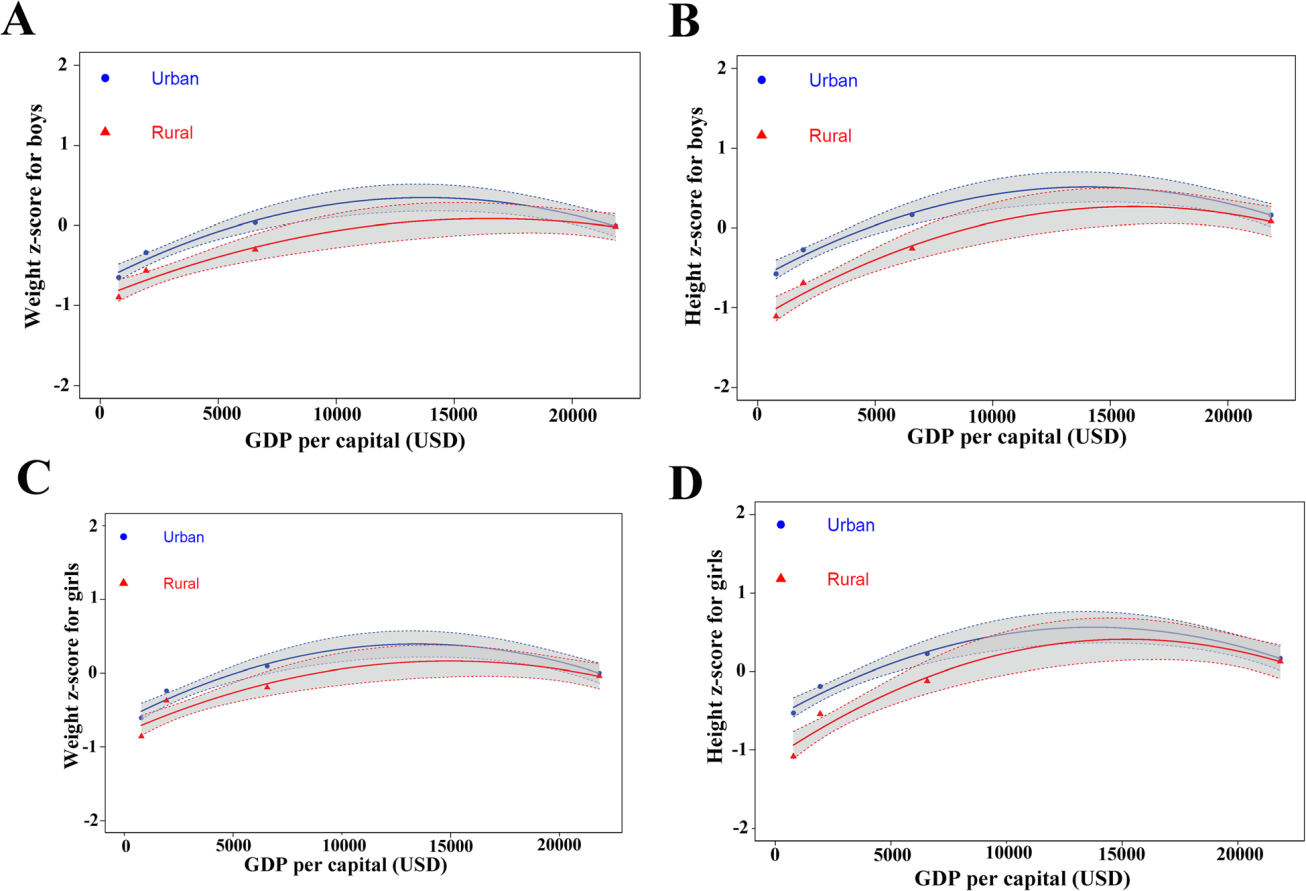
Association between growth z-score and GDP per capit. **a** The relationship between the GDP per capita and WAZ for boys. **b** The relationship between the GDP per capita and HAZ for boys. **c** The relationship between the GDP per capita and WAZ for girls. **d** The relationship between the GDP per capita and HAZ for girls

Furthermore, the association between economic development and z-scores were demonstrated in Fig. 3. With the development of GDP, the gap in boys’ weight between urban and rural areas narrowed, and disappeared when the GDP approached 25,000. It is displayed that the association pattern between economic growth and physical growth is an inversely U-shaped curve. Such trends can also be seen in the height of boys and girls, and weight of girls in urban and rural areas. These results indicated a trend of regional inequality in children’s physical growth, and these inequalities were associated with economic development.

### Trends in the prevalence of obesity and stunted growth in children in Guangzhou

The NESSOC surveys from 1996 to 2016 showed an increasing prevalence of obesity: the prevalence of obesity increased 10.2-fold in urban children, from 0.54% in 1996 to 5.53% in 2015, and 34-fold in rural children, from 0.23 to 7.82% (Supplementary Table 4). The prevalence of obesity in urban children increased rapidly during the three decades. In 2016, the prevalence of obesity in rural areas exceeded that in urban areas.

The prevalence of stunted growth in urban children was 2.18% in 1995 and decreased to 1.12% in 2015 (Supplementary Table 5). Among rural children, the prevalence was 2.96% in 1995 and decreased to 0.99% in 2015. The prevalence of stunted growth significantly decreased in both rural and urban area, with a greater reduction in rural areas (66.51% vs 48.73%).

### The association between children growth status and socioeconomic development

**Fig. 4:**
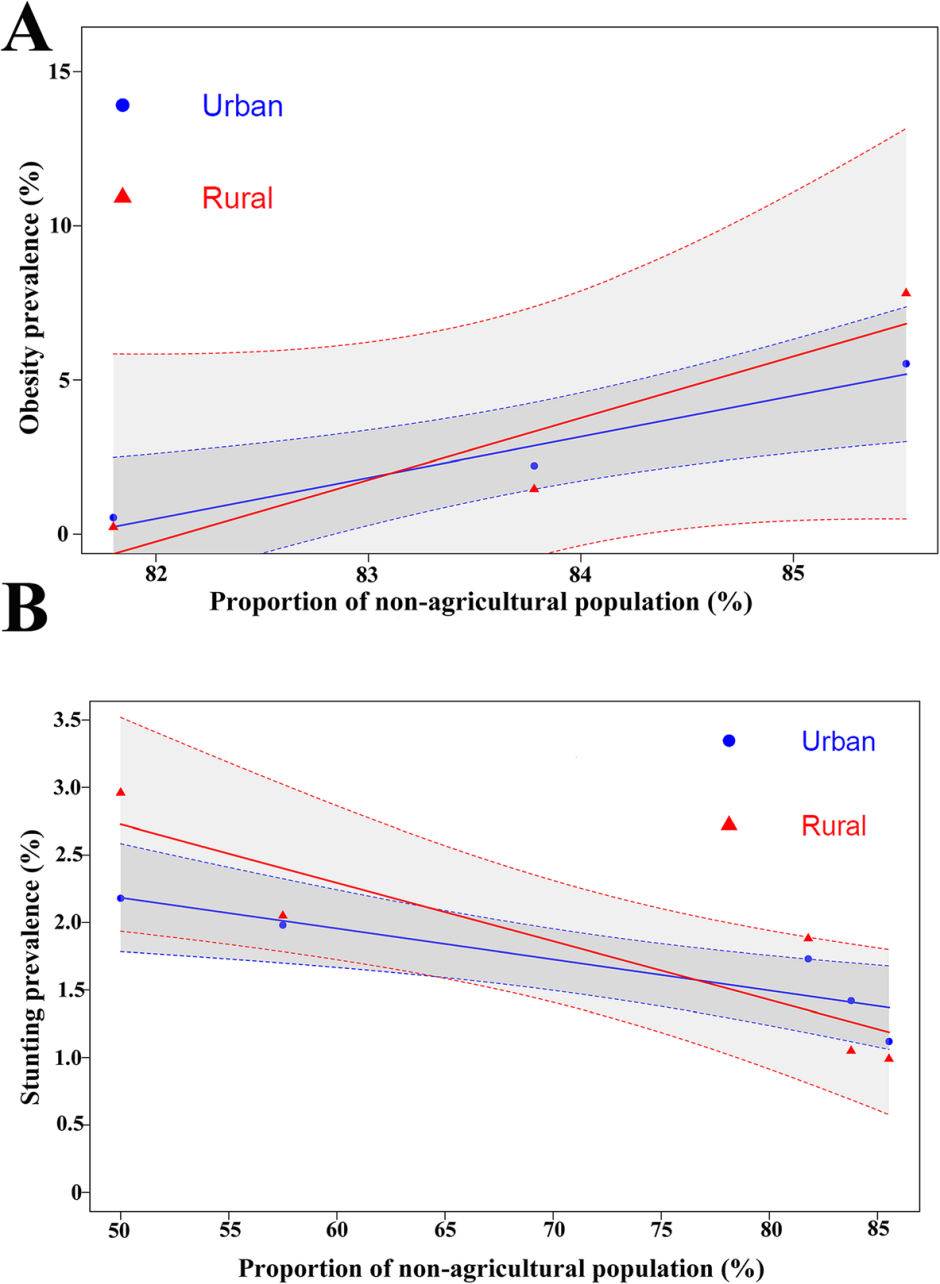
Association between urbanization rate and child nutritional status in 2015. **a** The relationship between the proportion of non-agricultural population and the prevalence of obesity in urban and rural areas. **b** The relationship between the proportion of non-agricultural population and the prevalence of stunted growth in urban and rural areas

**Fig. 5:**
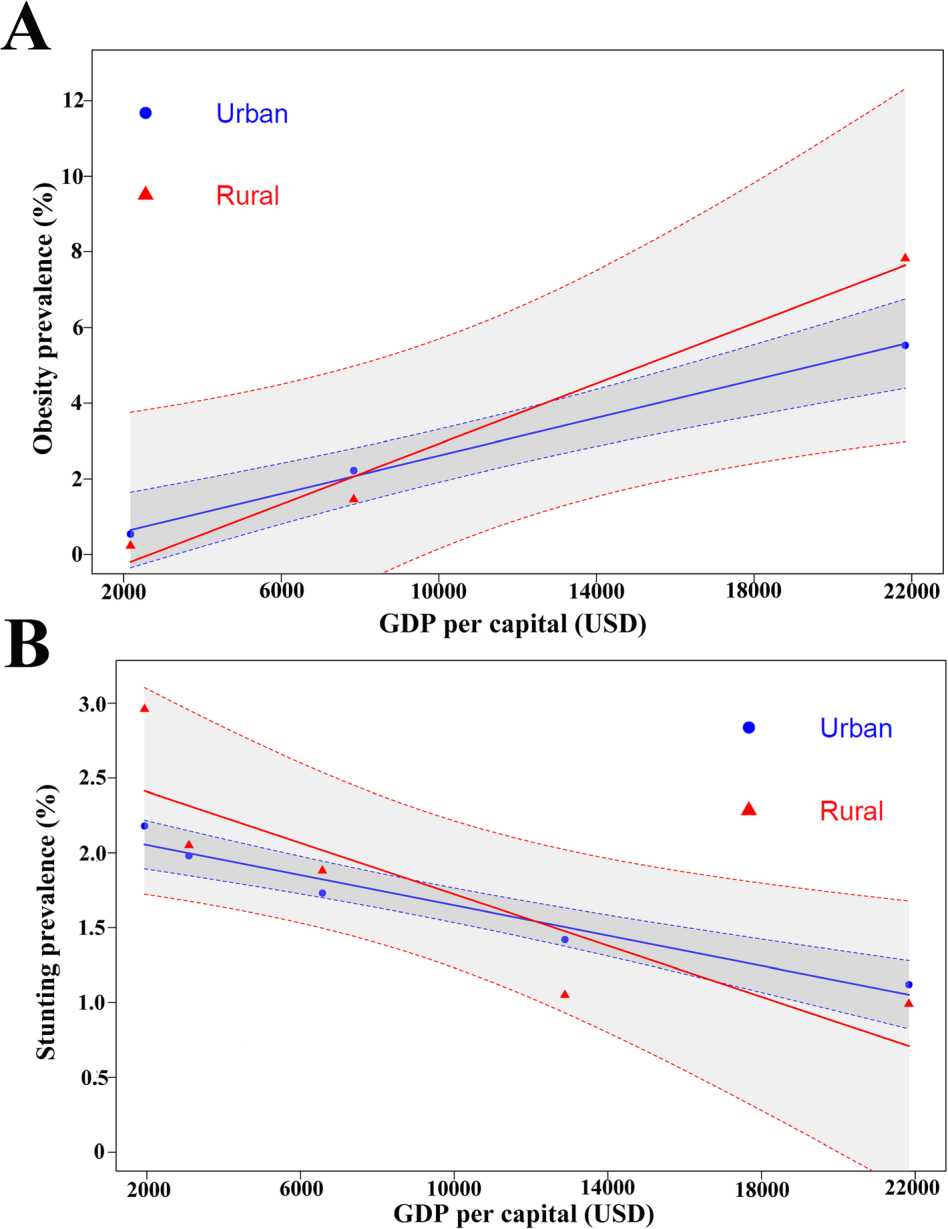
Association between GDP per capita and child nutritional status in 2015. **a** The relationship between the GDP per capita and the prevalence of obesity in urban and rural areas. **b** The relationship between the GDP per capita and the prevalence of stunted growth in urban and rural areas

The association between socioeconomic development and child growth were demonstrated in Fig. 4 and Fig. 5. The urbanization rate increased from 45.5% in 1985 to 85.5% in 2015. Strong positive associations were found between the prevalence of obesity and urbanization rate with estimated correlation coefficients *R* = 0.975 (*P* = 0.02) in urban children and *R* = 0.918 (*P* = 0.03) in rural children. And strong negative associations were also observed between the stunting prevalence and urbanization rate with *R* = -0.899 (*P* = 0.03) in urban area and *R* = -0.891 (*P* = 0.04) in rural area. Similar associations between changes in child nutritional status and GDP were detected, having the correlation coefficients *R* = 0.998 (*P* = 0.03) and *R* = 0.991 (*P* = 0.04) for the obesity prevalence in urban children, and *R* = -0.975 (*P* < 0.01) and *R* = -0.868 (*P* = 0.04) for the stunting prevalence in rural children.

## Discussion

In this study, we illustrated the secular trend in the physical development of children in urban and rural areas during 1985–2015 in Guangzhou. Results showed that the difference in height and weight decreased significantly from 1985 to 2015. In 2015, older children in rural areas weighted heavier than those of urban areas on average. Meanwhile, obesity kept increasing significantly in both areas, which seemed to be a more severe problem in rural areas. The prevalence of stunted growth decreased in both rural and urban areas. Economic development and urbanization were strongly associated with the prevalence of obesity and stunted growth.

As one of the largest cities in China, Guangzhou has underwent rapid urbanization, great improvement in economy and public health in the past three decades [21, 22]. GDP per capita in Guangzhou has increased from about 784 USD in 1985 to 21,835 USD in 2015. There has been a positive trend in the physical growth of Chinese children with socioeconomic progress [23]. In our study, we found that the physical growth of children in Guangzhou had increased significantly, and the inequality in their growth between urban and rural areas decreased in the past three decades. These results were consistent with previous studies [24–26]. NSPGDC literature from national data, showed that between 1985 and 2005, those who lived in a coastal city such as Guangzhou was taller on average, than those living in inland and small-medium size cities [27]. In the early stage of the study (1985–1995), the marked growth inequalities might result from poor economic status, lack of insurance and limited access to health services [28, 29]. In the middle stage (1995–2005), substantial urban/rural inequalities remained, in parallel with a sustained upward trend in physical growth. The decreasing health inequalities were probably due to the rapid economic development and the Basic Health Insurance Scheme policy in China [30, 31]. The gap between the physical growth of urban and rural children was further narrowed in the late stage (2005–2015). New health reforms, such as New Rural Co-operative Medical Care System (NRCMCS), were proposed during the same period. This basic public-health service project aimed to achieve health equity [32, 33], especially for children of migrant workers who lived in the suburbs of Guangzhou [34, 35]. In summary, economic development and expanding health care services largely decreased the urban/rural growth inequalities.

The differences in growth between urban and rural areas have narrowed over time. Generally, the physical growth status in children under 7 years old had been developed during the three decades both in rural and urban areas in Guangzhou. Besides, from the results of HAZ and WAZ in rural and urban areas and their differences in the three decades, we found that this gap of physical growth between rural and urban areas had become smaller since 2005. This changing pattern reflected that the physical growth rate of children in urban areas had decreased while increased in rural areas. These results were consistent with Xu’s cross-sectional surveys during 1985–2010, which reported that urban/rural differences in height were narrowed among children and adolescents aged 7–18 years in China [2]. In the Netherlands, the world’s tallest population has come to a halt after 150 years of secular growth, which may have reached the optimal height distribution [36]. Generally, economy affects children’s physical development by health care access, sanitary condition, nutrition and local infrastructure. Although income inequalities exist in urban and rural areas, adequate supply of nutrition, the improving economic and living conditions and better access to health care have gradually decreased the differences in growth rate of rural and urban children. Therefore, children in rural areas are catching up.

Another important nutritional issue was identified when physical growth of children was examined. The primary finding of our present study was that the prevalence of obesity in rural children increased 34-fold, which was more than that in urban children with better socioeconomic situations. Rapid urbanization in rural areas has led to new public health problems in children. It is widely reported that many changes in diet and physical activity are occurring simultaneously in the developing world and rural areas [37]. These changes were followed by a dramatic increase in obesity, hypertension and cardiovascular disease [38]. Urbanization and improving the economy in rural areas have enabled the rural population to purchase more processed foods and animal source foods [39, 40]. Also, only a few people in rural areas received high education, and instead most of them lacked the knowledge of maintaining a healthy life style and preventive strategies for chronic diseases [41–43]. A large proportion of the agricultural population and migrant population in the manufacturing industry lived in rural areas [44]. Therefore, there might be fewer effective behavioral interventions to prevent obesity [45].Our work highlighted an urgent need for developing appropriate interventions for children in the rural areas.

There were several strengths in our present study. Guangzhou represents an epitome of rapid urbanization and economic growth in China. Hence, it is important to examine the influence of its social changes over the past 30 years on child development. The results might predict the trends in other parts of China and even other developing countries that are going through dramatic social changes. In addition, the national surveillance data used in the present study was gathered throughout 30 successive years under strict quality control, and our study included a substantial proportion of the Chinese population in each period. However, several limitations existed in the present study. First, we lack the data of obesity and stunted growth from 1985 to 1995. We were not able to analyze or evaluate developmental indicators in depth in this period. Second, due to the limited number of data nodes in our survey, we were cautious in inferring the association between GDP, urbanization and growth, which might not be applicable for each individual. Furthermore, since the detailed demographic data of NSPGDC from 1985 to 2005 were not collected, we could not adjust for potentially important determinants of growth such as family income and parental education level in the present study. Therefore, how these determinants influence and to what extent they can explain the weight and height inequalities between urban and rural children remained unclear.

## Conclusions

In summary, our study showed great differences in the trend of physical growth between urban and rural children aged 0–6 years in Guangzhou. Although the economic growth and urbanization promoted the physical growth of rural children, emerging problems such as obesity is becoming a new challenge. Therefore, more public health services should be offered to migrant children and other disadvantaged situations should be improved. To eliminate health problems caused by child catch-up growth in rural areas, comprehensive intervention programs for early-childhood development should be promoted in rural areas. In particular, standardized child health-care services should be given early in life at community health service centers*.*

## Supplementary information

**Additional file 1: Figure S1.** Changes in physical growth of children < 7 years old in urban/rural areas in Guangzhou, 1985–2015. **Table S1.** Sample size of each subgroup in the NSPGDCs surveys, by sex-age. **Table S2.** Urban-rural height difference. **Table S3.** Urban-rural weight difference. **Table S4.** The prevalence of obesity in children < 7 years old in urban/rural areas in Guangzhou, 1996–2016. **Table S5.** The prevalence of Stunted growth in children < 7 years old in urban/rural areas in Guangzhou, 1995–2015.

## Data Availability

The data of growth and nutritional status used in the present study was extracted from the following 3 datasets: National Survey on the Physical Growth and Development of Children in the Nine Cities in China (NSPGDC), National Epidemiology Survey on Simple Obesity in Childhood (NESSOC), China National Maternal and Child Health Surveillance System (MCHSCN). We conducted data colloection of the above three suveys in Guangzhou. We were permitted by the national research group in China to analyse the data of our own collection and publish the results publicly. The economic data used in our present study were obtained from the Statistical Yearbook of the Guangzhou Statistics Bureau, which could be accessed in the official website (http://210.72.4.58/portal/queryInfo/statisticsYearbook/index).

## Abbreviations

NSPGDC: National Survey on the Physical Growth and Development of Children in the Nine Cities in China
NESSOC: National Epidemiology Survey on Simple Obesity in Childhood
MCHSCN: China National Maternal and Child Health Surveillance System
HAZ: height-for-age
WAZ: weight-for-age
GDP: Gross Domestic Product
