# Supplementary material for: Trends in urban/rural inequalities in physical growth among Chinese children over three decades of urbanization in Guangzhou: 1985–2015
Source: BMC Public Health. 2020 Jul 31;20:1190. doi: 10.1186/s12889-020-09239-7 (PMC7393843; doi:10.1186/s12889-020-09239-7)
Supplement: Supplementary file 1 — Additional file 1: Figure S1. Changes in physical growth of children < 7 years old in urban/rural areas in Guangzhou, 1985–2015. Table S1. Sample size of each subgroup in the NSPGDCs surveys, by sex-age. Table S2. Urban-rural height difference. Table S3. Urban-rural weight difference. Table S4. The prevalence of obesity in children < 7 years old in urban/rural areas in Guangzhou, 1996–2016. Table S5. The prevalence of Stunted growth in children < 7 years old in urban/rural areas in Guangzhou, 1995–2015. [file 12889_2020_9239_MOESM1_ESM.docx]

Supporting Information for

**Trends in urban/rural inequalities in physical growth among Chinese children over three decades**

Yan Hu, Weiqun Lin, Xuying Tan, Yu Liu, Yuqi Wen, Yanfei Xing, Ying Ma, Huiyan Liu, Yanyan Song, Jingjing Liang, Kin Bong Hubert Lam and Suifang Lin

**Correspondence:** Suifang Lin

Email: suifanglin@163.com


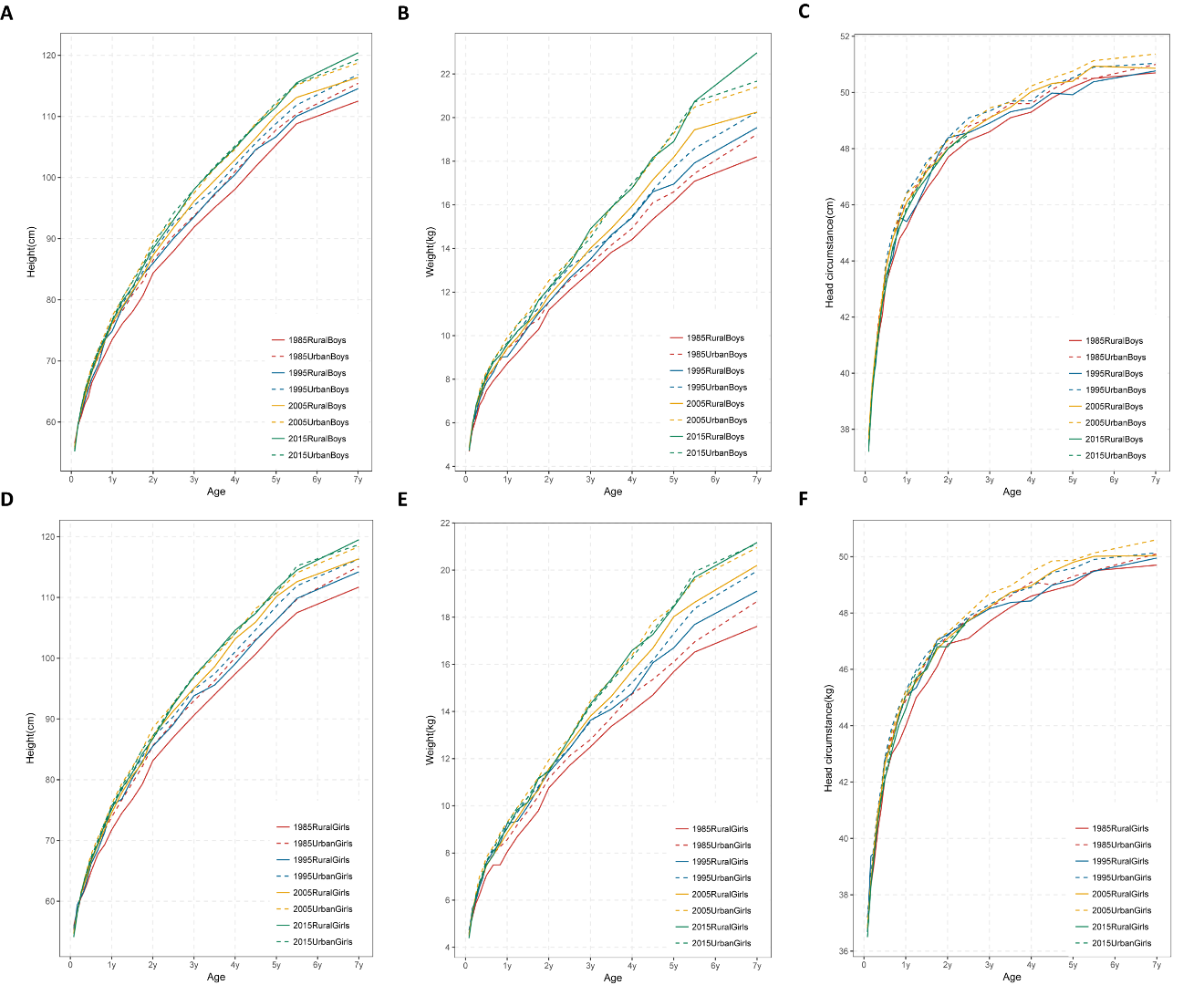


**Supplementary Figure 1. Changes in physical growth of children <7 years old in urban/rural areas in Guangzhou, 1985-2015.** (A-C Height, weight and head circumstance of rural and urban boys in consecutive surveys. D-F Height, weight and head circumstance of rural and urban girls in consecutive surveys.)

**Supplementary Table 1. Sample size of each subgroup in the NSPGDCs surveys, by sex-age.**

| **Age** | **Urban** | | | | | | | | **Rural** | | | | | | | |  |
| --- | --- | --- | --- | --- | --- | --- | --- | --- | --- | --- | --- | --- | --- | --- | --- | --- | --- |
|  | **1985** | | **1995** | | **2005** | | **2015** | | **1985** | | **1995** | | **2005** | | **2015** | |  |
|  | **Boys** | **Girls** | **Boys** | **Girls** | **Boys** | **Girls** | **Boys** | **Girls** | **Boys** | **Girls** | **Boys** | **Girls** | **Boys** | **Girls** | **Boys** | **Girls** |  |
| 1 mos | 200 | 200 | 200 | 200 | 181 | 179 | 215 | 234 | 150 | 150 | 155 | 159 | 185 | 189 | 196 | 229 | |
| 2 mos | 200 | 200 | 200 | 200 | 184 | 176 | 198 | 215 | 150 | 150 | 180 | 174 | 198 | 193 | 231 | 219 | |
| 3 mos | 200 | 200 | 200 | 200 | 183 | 178 | 193 | 204 | 150 | 150 | 154 | 187 | 190 | 189 | 236 | 227 | |
| 4 mos | 200 | 200 | 200 | 200 | 187 | 177 | 215 | 208 | 150 | 150 | 177 | 174 | 199 | 197 | 221 | 227 | |
| 5 mos | 200 | 200 | 200 | 200 | 178 | 191 | 161 | 186 | 150 | 150 | 197 | 195 | 184 | 186 | 231 | 237 | |
| 6 mos | 200 | 200 | 200 | 200 | 192 | 183 | 216 | 180 | 150 | 150 | 200 | 200 | 188 | 190 | 244 | 230 | |
| 8 mos | 200 | 200 | 200 | 200 | 191 | 192 | 185 | 192 | 150 | 150 | 200 | 200 | 176 | 165 | 214 | 213 | |
| 10 mos | 200 | 200 | 200 | 200 | 184 | 190 | 185 | 188 | 150 | 150 | 200 | 200 | 181 | 183 | 234 | 235 | |
| 12 mos | 200 | 200 | 200 | 200 | 187 | 184 | 194 | 175 | 200 | 200 | 200 | 200 | 193 | 179 | 251 | 228 | |
| 15 mos | 200 | 200 | 200 | 200 | 191 | 182 | 177 | 191 | 200 | 200 | 200 | 200 | 188 | 176 | 231 | 241 | |
| 18 mos | 200 | 200 | 200 | 200 | 184 | 177 | 193 | 197 | 200 | 200 | 200 | 200 | 178 | 171 | 237 | 239 | |
| 21 mos | 200 | 200 | 200 | 200 | 190 | 178 | 165 | 194 | 200 | 200 | 200 | 200 | 188 | 187 | 228 | 228 | |
| 2.0 yrs | 200 | 200 | 200 | 200 | 161 | 186 | 193 | 162 | 200 | 200 | 200 | 200 | 190 | 176 | 238 | 247 | |
| 2.5 yrs | 200 | 200 | 200 | 200 | 193 | 174 | 192 | 189 | 200 | 200 | 200 | 200 | 201 | 188 | 240 | 212 | |
| 3.0 yrs | 200 | 200 | 200 | 200 | 179 | 189 | 197 | 191 | 200 | 200 | 200 | 200 | 170 | 175 | 237 | 240 | |
| 3.5 yrs | 200 | 200 | 200 | 200 | 194 | 184 | 231 | 213 | 200 | 200 | 200 | 200 | 183 | 189 | 230 | 218 | |
| 4.0 yrs | 200 | 200 | 200 | 200 | 175 | 186 | 206 | 210 | 200 | 200 | 200 | 200 | 183 | 183 | 221 | 235 | |
| 4.5 yrs | 200 | 200 | 200 | 200 | 189 | 178 | 220 | 195 | 200 | 200 | 200 | 200 | 184 | 184 | 232 | 233 | |
| 5.0 yrs | 200 | 200 | 200 | 200 | 188 | 181 | 212 | 200 | 200 | 200 | 200 | 200 | 189 | 185 | 240 | 233 | |
| 5.5 yrs | 200 | 200 | 200 | 200 | 188 | 179 | 203 | 198 | 200 | 200 | 200 | 200 | 184 | 203 | 232 | 244 | |
| 6~7 yrs | 200 | 200 | 200 | 200 | 200 | 179 | 211 | 189 | 200 | 200 | 200 | 200 | 192 | 181 | 226 | 241 | |

**Supplementary Table 2. Urban-rural height difference**

|  | boys | | | | | | | | girls | | | | | | | |
| --- | --- | --- | --- | --- | --- | --- | --- | --- | --- | --- | --- | --- | --- | --- | --- | --- |
| year | 1985 | | 1995 | | 2005 | | 2015 | | 1985 | | 1995 | | 2005 | | 2015 | |
| Age | U-R difference | *P* value | U-R difference | *P* value | U-R difference | *P* value | U-R difference | *P* value | U-R difference | *P* value | U-R difference | *P* value | U-R difference | *P* value | U-R difference | *P* value |
| 1 mos | -0.60 | 0.008 | 0.40 | 0.073 | 0.45 | 0.057 | -0.10 | 0.604 | -0.60 | 0.008 | 0.84 | 0.000 | 0.11 | 0.617 | 0.10 | 0.590 |
| 2 mos | 0.00 | 1.000 | -0.27 | 0.255 | 0.14 | 0.529 | 0.10 | 0.631 | -0.40 | 0.071 | -0.80 | 0.004 | -0.29 | 0.195 | 0.10 | 0.598 |
| 3 mos | 0.50 | 0.043 | 0.66 | 0.006 | 0.21 | 0.331 | 0.00 | 1.000 | 0.00 | 1.000 | 0.21 | 0.366 | 0.39 | 0.059 | -0.10 | 0.593 |
| 4 mos | 0.90 | 0.001 | 1.02 | 0.000 | 1.04 | 0.000 | 0.40 | 0.059 | 0.90 | 0.000 | 1.36 | 0.000 | 0.64 | 0.003 | 0.20 | 0.349 |
| 5 mos | 2.00 | 0.000 | 0.66 | 0.005 | 0.93 | 0.000 | 0.20 | 0.402 | 1.60 | 0.000 | 1.07 | 0.000 | 1.13 | 0.000 | 0.30 | 0.149 |
| 6 mos | 1.70 | 0.000 | 1.69 | 0.000 | 1.07 | 0.000 | 0.70 | 0.001 | 2.20 | 0.000 | 0.92 | 0.000 | 1.13 | 0.000 | 0.50 | 0.038 |
| 8 mos | 1.80 | 0.000 | 2.14 | 0.000 | 1.18 | 0.000 | 0.40 | 0.098 | 1.50 | 0.000 | 1.66 | 0.000 | 1.19 | 0.000 | 0.40 | 0.110 |
| 10 mos | 2.10 | 0.000 | 0.47 | 0.132 | 1.08 | 0.000 | 0.40 | 0.103 | 2.40 | 0.000 | 0.82 | 0.001 | 1.06 | 0.000 | 0.40 | 0.082 |
| 12 mos | 2.50 | 0.000 | 1.71 | 0.000 | 1.22 | 0.000 | 0.20 | 0.431 | 2.10 | 0.000 | -0.37 | 0.325 | 1.49 | 0.000 | 0.40 | 0.138 |
| 15 mos | 2.10 | 0.000 | 0.67 | 0.074 | 1.30 | 0.000 | 0.50 | 0.085 | 2.20 | 0.000 | 1.56 | 0.000 | 1.51 | 0.000 | 0.40 | 0.185 |
| 18 mos | 2.80 | 0.000 | 0.67 | 0.086 | 1.77 | 0.000 | 0.90 | 0.004 | 2.70 | 0.000 | 0.89 | 0.012 | 1.40 | 0.000 | 0.60 | 0.031 |
| 21 mos | 2.30 | 0.000 | 0.45 | 0.195 | 2.21 | 0.000 | 0.40 | 0.163 | 2.90 | 0.000 | 0.66 | 0.057 | 2.26 | 0.000 | 0.80 | 0.010 |
| 2.0 yrs | 2.10 | 0.000 | 1.79 | 0.000 | 2.34 | 0.000 | 0.50 | 0.118 | 2.50 | 0.000 | 1.48 | 0.000 | 1.30 | 0.001 | 0.40 | 0.210 |
| 2.5 yrs | 2.50 | 0.000 | 2.46 | 0.000 | 1.39 | 0.000 | 1.10 | 0.004 | 2.40 | 0.000 | 1.31 | 0.000 | 1.20 | 0.002 | 0.30 | 0.429 |
| 3.0 yrs | 1.90 | 0.000 | 1.90 | 0.000 | 1.34 | 0.000 | 0.00 | 1.000 | 2.40 | 0.000 | 1.04 | 0.004 | 1.78 | 0.000 | 0.10 | 0.777 |
| 3.5 yrs | 2.00 | 0.000 | 0.81 | 0.028 | 1.96 | 0.000 | 0.20 | 0.610 | 2.30 | 0.000 | 1.83 | 0.000 | 1.64 | 0.000 | 0.00 | 1.000 |
| 4.0 yrs | 2.90 | 0.000 | 1.52 | 0.000 | 1.71 | 0.000 | 0.30 | 0.450 | 2.70 | 0.000 | 2.01 | 0.000 | 0.83 | 0.043 | -0.50 | 0.204 |
| 4.5 yrs | 2.60 | 0.000 | 1.07 | 0.005 | 2.41 | 0.000 | 0.10 | 0.803 | 2.40 | 0.000 | 1.73 | 0.000 | 2.22 | 0.000 | 0.20 | 0.604 |
| 5.0 yrs | 2.50 | 0.000 | 2.16 | 0.000 | 1.69 | 0.000 | 0.80 | 0.065 | 1.90 | 0.000 | 2.28 | 0.000 | 0.46 | 0.295 | -0.60 | 0.167 |
| 5.5 yrs | 1.60 | 0.000 | 1.83 | 0.000 | 2.04 | 0.000 | -0.20 | 0.651 | 2.20 | 0.000 | 2.07 | 0.000 | 1.46 | 0.000 | 0.70 | 0.134 |
| 6~7 yrs | 2.90 | 0.000 | 2.31 | 0.000 | 2.36 | 0.000 | -1.10 | 0.018 | 3.40 | 0.000 | 2.08 | 0.000 | 2.00 | 0.000 | -0.80 | 0.098 |

**Supplementary Table 3. Urban-rural weight difference**

|  | boys | | | | | | | | girls | | | | | | | |
| --- | --- | --- | --- | --- | --- | --- | --- | --- | --- | --- | --- | --- | --- | --- | --- | --- |
| year | 1985 | | 1995 | | 2005 | | 2015 | | 1985 | | 1995 | | 2005 | | 2015 | |
| Age | U-R difference | *P* value | U-R difference | *P* value | U-R difference | *P* value | U-R difference | *P* value | U-R difference | *P* value | U-R difference | *P* value | U-R difference | *P* value | U-R difference | *P* value |
| 1 mos | -0.22 | 0.000 | 0.07 | 0.256 | -0.01 | 0.878 | 0.01 | 0.856 | -0.13 | 0.024 | 0.16 | 0.003 | -0.01 | 0.862 | 0.00 | 1.000 |
| 2 mos | 0.08 | 0.268 | -0.02 | 0.752 | 0.05 | 0.486 | 0.03 | 0.650 | 0.05 | 0.454 | -0.10 | 0.110 | -0.05 | 0.413 | 0.05 | 0.372 |
| 3 mos | 0.13 | 0.104 | 0.18 | 0.012 | 0.03 | 0.690 | -0.09 | 0.226 | 0.07 | 0.333 | 0.11 | 0.082 | 0.06 | 0.385 | 0.07 | 0.280 |
| 4 mos | 0.23 | 0.007 | 0.16 | 0.043 | 0.30 | 0.000 | 0.11 | 0.168 | 0.32 | 0.000 | 0.21 | 0.003 | 0.32 | 0.000 | 0.03 | 0.679 |
| 5 mos | 0.33 | 0.000 | 0.00 | 1.000 | 0.38 | 0.000 | 0.12 | 0.175 | 0.32 | 0.000 | 0.21 | 0.006 | 0.27 | 0.001 | 0.11 | 0.149 |
| 6 mos | 0.52 | 0.000 | 0.34 | 0.000 | 0.25 | 0.005 | 0.14 | 0.106 | 0.42 | 0.000 | 0.01 | 0.899 | 0.36 | 0.000 | 0.18 | 0.034 |
| 8 mos | 0.52 | 0.000 | 0.44 | 0.000 | 0.41 | 0.000 | 0.09 | 0.357 | 0.46 | 0.000 | 0.17 | 0.045 | 0.35 | 0.000 | 0.21 | 0.017 |
| 10 mos | 0.58 | 0.000 | 0.11 | 0.233 | 0.42 | 0.000 | 0.21 | 0.028 | 0.77 | 0.000 | 0.18 | 0.038 | 0.49 | 0.000 | 0.16 | 0.079 |
| 12 mos | 0.69 | 0.000 | 0.61 | 0.000 | 0.51 | 0.000 | 0.08 | 0.392 | 0.52 | 0.000 | -0.19 | 0.111 | 0.44 | 0.000 | 0.21 | 0.026 |
| 15 mos | 0.55 | 0.000 | 0.48 | 0.000 | 0.60 | 0.000 | 0.31 | 0.002 | 0.50 | 0.000 | 0.38 | 0.000 | 0.44 | 0.000 | 0.10 | 0.319 |
| 18 mos | 0.66 | 0.000 | 0.43 | 0.000 | 0.51 | 0.000 | 0.30 | 0.008 | 0.55 | 0.000 | 0.38 | 0.001 | 0.41 | 0.001 | 0.19 | 0.068 |
| 21 mos | 0.45 | 0.000 | 0.22 | 0.053 | 0.72 | 0.000 | -0.06 | 0.610 | 0.62 | 0.000 | 0.06 | 0.613 | 0.53 | 0.000 | 0.03 | 0.797 |
| 2.0 yrs | 0.38 | 0.001 | 0.52 | 0.000 | 0.70 | 0.000 | 0.02 | 0.882 | 0.41 | 0.000 | 0.14 | 0.230 | 0.38 | 0.005 | 0.02 | 0.861 |
| 2.5 yrs | 0.45 | 0.000 | 0.51 | 0.000 | 0.55 | 0.000 | 0.20 | 0.210 | 0.41 | 0.001 | 0.02 | 0.879 | 0.24 | 0.109 | -0.01 | 0.948 |
| 3.0 yrs | 0.38 | 0.006 | 0.36 | 0.007 | 0.70 | 0.000 | -0.41 | 0.013 | 0.31 | 0.023 | -0.06 | 0.645 | 0.65 | 0.000 | -0.09 | 0.571 |
| 3.5 yrs | 0.33 | 0.012 | -0.07 | 0.610 | 0.94 | 0.000 | -0.01 | 0.957 | 0.37 | 0.007 | 0.29 | 0.042 | 0.72 | 0.000 | -0.09 | 0.608 |
| 4.0 yrs | 0.51 | 0.001 | 0.06 | 0.705 | 0.81 | 0.000 | 0.21 | 0.319 | 0.73 | 0.000 | 0.46 | 0.002 | 0.66 | 0.001 | -0.31 | 0.137 |
| 4.5 yrs | 0.76 | 0.000 | 0.07 | 0.653 | 0.91 | 0.000 | -0.11 | 0.661 | 0.66 | 0.000 | 0.11 | 0.503 | 1.12 | 0.000 | 0.17 | 0.443 |
| 5.0 yrs | 0.43 | 0.007 | 0.77 | 0.000 | 1.10 | 0.000 | 0.46 | 0.071 | 0.42 | 0.017 | 0.60 | 0.001 | 0.44 | 0.057 | 0.06 | 0.814 |
| 5.5 yrs | 0.36 | 0.039 | 0.66 | 0.000 | 1.04 | 0.000 | -0.02 | 0.953 | 0.43 | 0.013 | 0.68 | 0.001 | 0.96 | 0.000 | 0.24 | 0.443 |
| 6~7 yrs | 1.03 | 0.000 | 0.71 | 0.003 | 1.15 | 0.000 | -1.30 | 0.001 | 1.06 | 0.000 | 0.85 | 0.000 | 0.75 | 0.008 | -0.05 | 0.878 |

Supplementary Table 4. The prevalence of obesity in children <7 years old

in urban/rural areas in Guangzhou, 1996-2016.

| Year | Stunted growth | | |
| --- | --- | --- | --- |
|  | Urban | Rural | Absolute difference |
| 1996 | 0.54 | 0.23 | 0.31 |
| 2006 | 2.22 | 1.46 | 0.76 |
| 2016 | 5.53 | 7.82 | -2.29 |

Supplementary Table 5. The prevalence of Stunted growth in children <7 years old

in urban/rural areas in Guangzhou, 1995-2015.

| Year | Stunted growth | | |
| --- | --- | --- | --- |
|  | Urban | Rural | Absolute difference |
| 1995 | 2.18 | 2.96 | -0.78 |
| 2000 | 1.98 | 2.05 | -0.07 |
| 2005 | 1.73 | 1.88 | -0.15 |
| 2010 | 1.42 | 1.05 | 0.37 |
| 2015 | 1.12 | 0.99 | 0.13 |
